# Emergence of multidrug-resistant *Providencia rettgeri* isolates co-producing NDM-1 carbapenemase and PER-1 extended-spectrum β-lactamase causing a first outbreak in Korea

**DOI:** 10.1186/s12941-018-0272-y

**Published:** 2018-05-05

**Authors:** Saeam Shin, Seok Hoon Jeong, Hyukmin Lee, Jun Sung Hong, Min-Jeong Park, Wonkeun Song

**Affiliations:** 1 Department of Laboratory Medicine, Hallym University College of Medicine, Seoul, South Korea; 2 Department of Laboratory Medicine and Research Institute for Antimicrobial Resistance, Yonsei University College of Medicine, Seoul, South Korea

**Keywords:** *Providencia rettgeri*, Outbreak, Urinary tract infection, NDM-1, PER-1

## Abstract

**Background:**

Nosocomial outbreak due to carbapenem-resistant Enterobacteriaceae has become serious challenge to patient treatment and infection control. We describe an outbreak due to a multidrug-resistant *Providencia rettgeri* from January 2016 to January 2017 at a University Hospital in Seoul, Korea.

**Methods:**

A total of eight non-duplicate *P. rettgeri* isolates were discovered from urine samples from eight patients having a urinary catheter and admitted in a surgical intensive care unit. The β-lactamase genes were identified using polymerase chain reaction and direct sequencing, and strain typing was done with pulsed-field gel electrophoresis (PFGE).

**Results:**

All isolates showed high-level resistance to extended-spectrum cephalosporins, aztreonam, meropenem, ertapenem, ciprofloxacin, and amikacin. They harbored the *bla*_NDM-1_ carbapenemase and the *bla*_PER-1_ type extended-spectrum β-lactamases genes. PFGE revealed that all isolates from eight patients were closely related strains.

**Conclusions:**

The 13-month outbreak ended following reinforcement of infection control measures, including contact isolation precautions and environmental disinfection. This is the first report of an outbreak of a *P. rettgeri* clinical isolates co-producing NDM-1 and PER-1 β-lactamase.

## Background

The genus *Providencia* comprises part of the natural human gut flora but may also cause infections, including travelers’ diarrhea, urinary tract infections, and other nosocomial infections [1]. Treatment of these infections is challenging because *Providencia rettgeri* strains are intrinsically resistant to many antimicrobials including ampicillin, first generation cephalosporins, polymyxins and tigecycline [2]. Furthermore, in recent years *P. rettgeri* has become increasingly important because of the emergence of carbapenemase-producing strains [3, 4]. Carbapenemases are enzymes known to hydrolase almost all types of β-lactams [5]. The New Delhi metallo-β-lactamase (NDM-1) has been firstly identified in 2009 in a Swedish patient who had been previously hospitalized in New Delhi, India [6]. The first occurrence of NDM-1 producers was reported in clinical isolates of *P. rettgeri* in Israel in 2013 [7]. Since then, other cases have been reported in Mexico, Brazil, Argentina, Ecuador, Canada, and Nepal [3, 4, 8–13].

PER-1 enzyme is belong to class A extended-spectrum β-lactamases (ESBLs) and firstly discovered in a plasmid of *Pseudomonas aeruginosa* in France [14]. Later, it has also found among several Gram-negative species including *Acinetobacter baumannii*, *Salmonella enterica* serovar Typhimurium, and also in *P. rettgeri* [15, 16]. PER-1 is widely spread in Turkey, however, high prevalence of PER-1 ESBL in *A. baumannii* has been reported in Korea [17].

Here, we report the first outbreak of multidrug-resistant *P. rettgeri* strain co-producing NDM-1 and PER-1 in Korea.

## Materials and methods

### Patients and bacterial isolates

From January 2016 to January 2017, a total of eight *P. rettgeri* isolates from eight patients were included in this study. Bacterial identification was done with a Vitek-MS (bioMérieux, Marcy I’Etoile, France). Medical records of the patients were retrospectively reviewed. This study protocol was approved by the hospital institutional review board.

### Antimicrobial susceptibility testing

Minimum inhibitory concentrations (MICs) for cefotetan, cefotaxime, ceftazidime, cefepime, ertapenem, imipenem, meropenem, aztreonam, amikacin, ciprofloxacin, gentamicin, and tigecycline were determined using Etest strips (bioMérieux) on the Mueller–Hinton agar (Becton–Dickinson, Sparks, MD, USA). Colistin MIC was determined by broth microdilution. When available, antimicrobial susceptibility was interpreted based on the Clinical and Laboratory Standards Institute (CLSI) guideline [18]. For tigecycline and colistin, the European Committee for Antimicrobial Susceptibility Testing (EUCAST) criteria were used [19].

### Detection of β-lactamase genes

The carbapenemase genes and ESBL genes were detected using specific PCR primers (Table 1) [20–27]. Amplified products were directly sequenced on the ABI 3730xl automatic sequencer (Applied Biosystems, Foster City, CA, USA) using the same primer pair. The sequences obtained were compared to those in GenBank (www.ncbi.nlm.nih.gov/GenBank) using the BLAST program (www.ncbi.nlm.nih.gov/BLAST/).

**Table 1:** Primers used in this study for identifying antimicrobial resistance genes

Classification	Primer	Target	Nucleotide sequence, 5′ to 3′	Product size, bp	References
Class A β lactamases	VEB-1F	*bla*_VEB_	CGACTTCCATTTCCCGATGC	642	[20]
	VEB-1R		GGACTCTGCAACAAATACGC		
	PER-1F	*bla*_PER-1_	ATGAATGTCATTATAAAAGCT	927	[20]
	PER-1R		TTAATTTGGGCTTAGGG		
	CTX-M-1F	*bla*_CTX-M-1_	GCAGCACCAGTAAAGTGATGG	591	[21]
	CTX-M-1R		GCTGGGTGAAGTAAGTGACC		
	CTX-M-825F	*bla*_CTX-M-8_	CGCTTTGCCATGTGCAGCACC	307	[22]
	CTX-M-825R		GCTCAGTACGATCGAGCC		
	CTX-M-914F	*bla*_CTX-M-9_	GCTGGAGAAAAGCAGCGGAG	474	[22]
	CTX-M-914R		GTAAGCTGACGCAACGTCTG		
	SHV-OS5	*bla*_SHV_	TTATCTCCCTGTTAGCCA	797	[23]
	SHV-OS6		GATTTGCTGAATTCGCTC		
	TEM-A	*bla*_TEM_	TAAAATTCTTGAAGACG	1074	[23]
	TEM-B		TTACCAATGCTTAATCA		
	KPC-F	*bla*_KPC_	ATGTCACTGTATCGCCGTCT	893	[24]
	KPC-R		TTTTCAGAGCCTTACTGCCC		
Class B β lactamases	VIM-F	*bla*_VIM_	GATGGTGTTTGGTCGCATA	390	[25]
	VIM-R		CGAATGCGCAGCACCAG		
	IMP-F	*bla*_IMP_	GGAATAGAGTGGCTTAATTC	232	[26]
	IMP-R		TCGGTTTAATAAAACAACCACC		
	NDM-1-F	*bla*_NDM-1_	CAATATTATGCACCCGGTCG	726	[27]
	NDM-1-R		ATCATGCTGGCCTTGGGGAA		
Class D β lactamases	OXA-10F	*bla*_OXA-10_	TATCGCGTGTCTTTCGAGTA	760	[20]
	OXA-10R		TTAGCCACCAATGATGCCC		
	OXA-F	*bla*_OXA-48_	GCGTGGTTAAGGATGAACAC	438	[26]
	OXA-R		CATCAAGTTCAACCCAACCG		

### Pulsed-field gel electrophoresis

The bacterial genetic relatedness was evaluated by Pulsed-field gel electrophoresis (PFGE). Genomic DNA was digested with *SfiI* enzyme, and DNA fragments were separated on a CHEF-DRII System (Bio-Rad, Hercules, CA, USA). A lambda ladder (Bio-Rad) was used as a DNA size marker. The band patterns were analyzed using UVIband/Map software (UVItech Ltd., Cambridge, UK) and the dendrograms were generated based on the unweighted pair group method using arithmetic averages from the Dice coefficient. Isolates that exhibited a PFGE profile with more than 90% similarity (pulsotype) were considered as closely related strains.

## Results

**Fig. 1:**
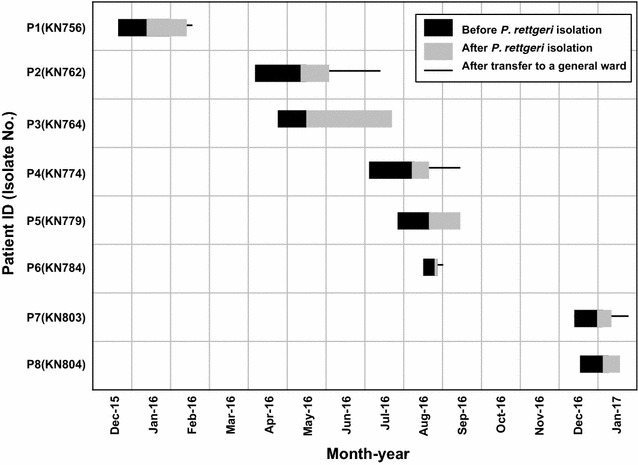
Time course of the outbreak by multidrug-resistant *Providencia rettgeri*. Black bars indicate the pre-infection period and gray bars the post-infection period in the surgical intensive care unit. Solid lines indicate the period during patients was hospitalized in a general ward

**Fig. 2:**
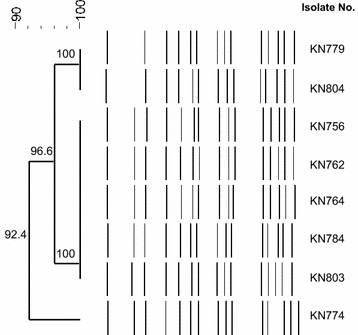
Pulsed-field gel electrophoresis patterns of *Providencia rettgeri* clinical isolate co-producing NDM-1 and PER-1. All eight isolates from the outbreak were closely related strains

The characteristics of these patients and antimicrobial susceptibility patterns of *P. rettgeri* isolates were summarized in Table 2. In total, eight *P. rettgeri* isolates were recovered from urine samples of eight patients admitted in a surgical intensive care unit (SICU). All patients were admitted to a SICU from hospitalization and had a urinary catheter. The median days of the SICU stay before *P. rettgeri* isolation was 21.5 days (range, 8–38 days) (Fig. 1). All patients except one (P5) were recovered and discharged during the outbreak. A patient (P5) died following *Enterococcus faecalis* bacteremia. All *P. rettgeri* isolates showed similar antibiogram with high MIC levels to various classes of antimicrobial agents tested (cefotetan, cefotaxime, ceftazidime, cefepime, azteronam, meropenem, ertapenem, ciprofloxacin, amikacin, and tigecycline). Imipenem MICs were 0.5–4 μg/mL (6/8 susceptible isolates, 1/8 intermediate isolate, and 1/8 resistant isolate) and gentamicin MICs were 8–16 μg/mL (4/8 intermediate isolates and 4/8 resistant isolates). Molecular testing revealed that all the *P. rettgeri* isolates were positive for *bla*_NDM-1_ and *bla*_PER-1_. No amplicons were observed for the other primer pairs for *bla*_VEB_, *bla*_CTX-M-1_, *bla*_CTX-M-8_, *bla*_CTX-M-9_, *bla*_SHV_, *bla*_TEM_, *bla*_KPC_, *bla*_VIM_, *bla*_IMP_, *bla*_OXA-10_, and *bla*_OXA-48_. PFGE revealed that all isolates closely related one pulsotype with > 90% similarity (Fig. 2). The eight isolates had the three kinds of dendrogram patterns.

**Table 2:** Clinical characteristics of the outbreak cases and antimicrobial susceptibility profiles of *Providencia rettgeri* isolates

Patient ID	P1	P2	P3	P4	P5	P6	P7	P8
Isolate no.	KN756	KN762	KN764	KN774	KN779	KN784	KN803	KN804
Sex/age (year)	M/63	M/50	M/52	M/66	F/75	M/81	F/40	M/53
Diagnosis	Brain hemorrhage	Deep neck infection	Central nervous system infection	Bladder cancer	Pneumonia	Pneumonia	Brain hemorrhage	Brain hemorrhage
Comorbidities	–	Diabetes mellitus	–	–	Cerebral infarction	Diabetes mellitus	–	–
Outcome	Survival	Survival	Survival	Survival	Death	Survival	Survival	Survival
Hospital admission date	18-Dec-15	05-Apr-16	28-Apr-16	01-Jul-16	28-Jul-16	14-Aug-16	16-Dec-16	19-Dec-16
*P. rettgeri* collection date	11-Jan-16	09-May-16	19-May-16	08-Aug-16	19-Aug-16	22-Aug-16	30-Dec-16	05-Jan-17
Antimicrobial agents used before *P. rettgeri* isolation (days)	Colistin (13), piperacillin-tazobactam (8), teicoplanin (11)	Colistin (21), metronidazole (10), piperacillin-tazobactam (10), ampicillin-sulbactam (3), teicoplanin (20), netilmicin (5), levofloxacin (9)	Colistin (13), piperacillin-tazobactam (3), vancomycin (8), teicoplanin (13), meropenem (7)	Ceftriaxone (6), tigecycline (4), doripenem (7), piperacillin-tazobactam (18), flomoxef (3), teicoplanin (5)	Metronidazole (10), moxifloxacin (6), piperacillin-tazobactam (2), teicoplanin (2)	Piperacillin-tazobactam (5), ampicillin-sulbactam (3)	Ceftriaxone (3)	Ceftriaxone (8)
MIC (μg/mL)								
Cefotetan	> 256	> 256	> 256	> 256	> 256	> 256	> 256	> 256
Cefotaxime	> 32	> 32	> 32	> 32	> 32	> 32	> 32	> 32
Ceftazidime	> 256	> 256	> 256	> 256	> 256	> 256	> 256	> 256
Cefepime	> 256	> 256	> 256	> 256	> 256	> 256	> 256	> 256
Aztreonam	> 256	> 256	> 256	> 256	> 256	> 256	> 256	> 256
Imipenem	0.5	0.5	2	4	0.5	1	0.5	0.5
Meropenem	> 32	> 32	> 32	> 32	> 32	> 32	> 32	> 32
Ertapenem	> 32	> 32	> 32	> 32	> 32	> 32	> 32	> 32
Ciprofloxacin	> 32	> 32	> 32	> 32	> 32	> 32	> 32	> 32
Amikacin	> 256	> 256	> 256	> 256	> 256	> 256	> 256	> 256
Gentamicin	8	16	16	16	8	16	8	8
Tigecycline	4	4	4	8	4	8	8	8
Colistin	2	2	8	2	2	64	4	4

*MIC* minimum inhibitory concentration

## Discussion

In the present study we reported and characterized an outbreak of *bla*_NDM-1_ and *bla*_PER-1_ carrying *P. rettgeri*. All patients were admitted to the same SICU and had a urinary catheter. *P. rettgeri* is well known to be isolated from urine of hospitalized and catheterized patients [16]. Although periods of hospitalization of our patients were not completely overlapping, PFGE revealed that all isolates were closely related. This suggests clonal cross-transmission of this strain in the SICU, and there is a possibility of transmission between patients and medical personnel by hand colonization or by environmental contamination. Infection control measures were reinforced in the SICU to include extensive environmental disinfection, active screening for carbapenemase-producing Enterobacteriaceae, and exhaustive contact isolation precautions. The outbreak did not eradicate in a short time, but the outbreak was eventually interrupted in January 2017.

Carbapenem resistance in Enterobacteriaceae has become a major public health challenge [28]. While carbapenem is a drug of choice for treatment of Enterobacteriaceae producing ESBL and plasmid-mediated AmpC cephalosporinase, production of carbapenemase in Enterobacteriaceae can be emerged. Carbapenemase gene is important due to its potential transferability to other species, by plasmids and transposons [28]. NDM-1 encoding plasmids are diverse and can also carry other antimicrobial resistance genes, including carbapenemase genes, ESBL genes, plasmid-mediated cephalosporinase genes, and aminoglycoside resistance genes [28, 29]. Among these, most ESBLs found with NDM-1 have been reported to be as CTX-M-15 type [29, 30]. Until now, this is the first report of Enterobacteriaceae co-carrying NDM-1 and PER-1 type ESBL. Although the NDM-1 enzyme is known to inactivate all β-lactams except aztreonam [6], our *P. rettgeri* isolates showed high MIC to aztreonam, possibly due to production of PER-1 type ESBL. The range of MIC to imipenem revealed 0.5–4 μg/mL. Imipenem MICs for *Providencia* spp. tend to be higher (e.g., MICs in the intermediate or resistant range) naturally. These isolates may have elevated imipenem MICs by mechanisms other than production of carbapenemases [18].

It is known that the multidrug-resistant bacteria have superior ability to survive and spread successfully in a hospital environment. In addition, the patient’s risk factor is also responsible for the nosocomial transmission of multidrug-resistant bacteria. Patient’s underlying disease, exposure to antimicrobial agents, and history of having invasive procedures are known as risk factors for the acquisition of carbapenem-resistant Enterobacteriaceae [28]. This outbreak persisted for 13 months, although the prompt infection control strategy was initiated after recognition of the first few cases. Because ICU admission patients often have one or more of risk factors, so it could be very difficult to eradicate once the outbreak occurs.

In conclusion, we report an alarming outbreak of high-level of multidrug-resistant *P. rettgeri* isolates co-producing NDM-1 and PER-1 β-lactamases. Infection prevention and control efforts should be continuously made to prevent nosocomial transmission of these threatening bacteria.
